# Interaction of DNA demethylase and histone methyltransferase upregulates Nrf2 in 5-fluorouracil-resistant colon cancer cells

**DOI:** 10.18632/oncotarget.9745

**Published:** 2016-05-31

**Authors:** Kyoung Ah Kang, Mei Jing Piao, Yea Seong Ryu, Hee Kyoung Kang, Weon Young Chang, Young Sam Keum, Jin Won Hyun

**Affiliations:** ^1^ School of Medicine, Jeju National University, Jeju 63243, Republic of Korea; ^2^ Department of Biochemistry, College of Pharmacy, Dongguk University, Goyang 10326, Republic of Korea

**Keywords:** Nrf2 transcription factor, DNA demethylase, histone methyltransferase, 5-fluorouracil-resistance, oxidative stress

## Abstract

We recently reported that DNA demethylase ten-eleven translocation 1 (TET1) upregulates nuclear factor erythroid 2-related factor 2 (Nrf2) in 5-fluorouracil-resistant colon cancer cells (SNUC5/5-FUR). In the present study, we examined the effect of histone modifications on Nrf2 transcriptional activation. Histone deacetylase (HDAC) and histone acetyltransferase (HAT) were respectively decreased and increased in SNUC5/5-FUR cells as compared to non-resistant parent cells. Mixed-lineage leukemia (MLL), a histone methyltransferase, was upregulated, leading to increased trimethylation of histone H3 lysine 4, while G9a was downregulated, leading to decreased dimethylation of histone H3 lysine 9. siRNA-mediated MLL knockdown decreased levels of Nrf2 and HO-1 to a greater extent than did silencing HAT1. Host cell factor 1 (HCF1) was upregulated in SNUC5/5-FUR cells, and we observed interaction between HCF1 and MLL. Upregulation of O-GlcNAc transferase (OGT), an activator of HCF1, was also associated with HCF1-MLL interaction. In SNUC5/5-FUR cells, a larger fraction of OGT was bound to TET1, which recruits OGT to the Nrf2 promoter region, than in SNUC5 cells. These findings indicate that SNUC5/5-FUR cells are under oxidative stress, which induces expression of histone methylation-related proteins as well as DNA demethylase, leading to upregulation of Nrf2 and 5-FU resistance.

## INTRODUCTION

Histone modifications including methylation, acetylation, ubiquitination, and phosphorylation regulate gene expression programs. In particular, the mixed-lineage leukemia (MLL) family of histone methyltransferases regulates gene expression by methylating lysine 4 of histone H3 (H3K4), which is associated with an active chromatin state [1]. Histone-lysine N-methyltransferase, SET, or MLL acts as the catalytic subunit of the protein complexes associated with the SET/COMPASS complex or MLL/COMPASS-like complex [2]. These subunits aid in complex assembly and recruitment to targets, and modulate the methyltransferase activity of the SET domain-containing subunits [1, 3]. For example, host cell factor 1 (HCF1) is a component of the H3K4 methyltransferase SET/COMPASS complex and is important for its integrity [4].

The ten-eleven translocation (TET) family proteins, including TET1, TET2, and TET3, catabolize the oxidation of 5-methylcytosine to 5-hydroxylmethylcytosine, 5-formylcytosine, and 5-carboxylcytosine, resulting in the formation of cytosine [5]. TET proteins have been implicated in genome-wide DNA methylation control, gene expression regulation, cellular differentiation, and cancer development [6–8].

DNA methylation is generally associated with gene silencing, while DNA demethylation via TET leads to transcriptional activation. Recent studies suggest that the interaction of TET1 with O-GlcNAc transferase (OGT) stabilizes TET1 binding to target promoters [6, 9]. Genome-wide localization analyses show enrichment of TET1 on regulatory regions marked by H3K4 trimethylation (H3K4Me3) [10, 11]. Furthermore, TET2 and TET3 regulate GlcNAcylation and H3K4 methylation through OGT and SET/COMPASS [4]. This suggests that in addition to its role in reducing DNA methylation, the TET-OGT interaction recruits proteins required to establish a high H3K4Me3 chromatin environment

Oxidative stress is involved in most chronic diseases including cancer. Interestingly, epigenetic modification of DNA and histones is modulated by oxidative stress [12]. Recently, we reported that nuclear factor erythroid 2-related factor 2 (Nrf2), a major transcription factor for antioxidant enzymes, is highly expressed in 5-fluorouracil (5-FU)-resistant cells under oxidative stress through the DNA demethylating function of TET1 [13].

In the present study, we aimed to determine whether histone methyl-modifications are involved in the modulation of Nrf2 expression in 5-FU-resistant cells and the role of TET1 in histone methyl-modifications. This report is the first to examine the relationship between histone methyltransferase and DNA demethylase and modulation of Nrf2 expression.

## RESULTS

### Expression of Nrf2 in chemo-resistant cancer cells

**Figure 1:**
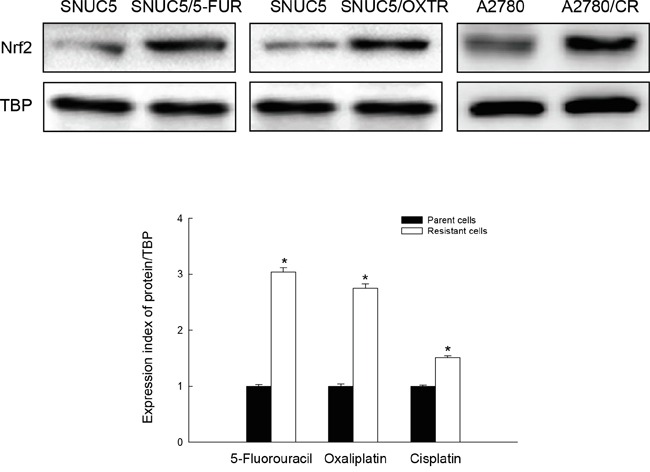
Nrf2 protein level in chemo-resistant cancer cells The nuclear Nrf2 protein level in SNUC5 and SNUC5/5-FUR, SNUC5 and SNUC5/OXTR, A2780 and A2780/CR were assessed using Western blot analysis. TBP antibody was used as loading control for nuclear fraction. Densito-metric quantification of band intensity was measured and normalized relative to the band intensity of the TBP loading control. *Significantly different from parent cells respectively (p<0.05).

Previously, we reported that Nrf2 expression was higher in 5-FU-resistant colon cancer cells (SNUC5/5-FUR) than parent colon cancer cells (SNUC5) [14]. Here, in addition to SNUC5/5-FUR, we determined that Nrf2 expression was higher in oxaliplatin resistant SNUC5 cells (SNUC5/OXTR) and cisplatin resistant ovarian cancer cells (A2780/CR) than in parental SNUC5 and A2780 cells, respectively (Figure 1). These data link Nrf2 to chemo-resistance in cancer cells, and led us to select SNUC5/5-FUR cells for further study.

### Expression of histone modification-related proteins in SNUC5 and SNUC5/5-FUR cells

**Figure 2:**
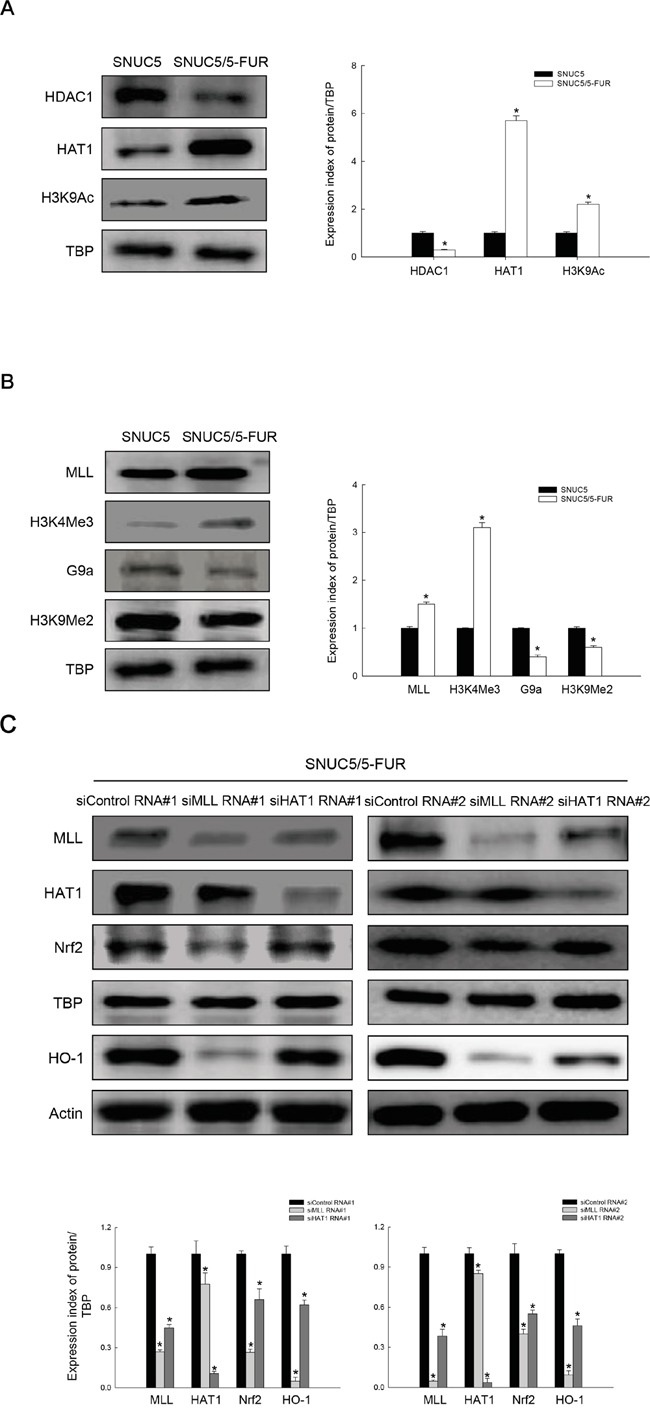
Expression of histone modification-related proteins in SNUC5 and SNUC5/5-FUR **A.** Expression patterns of HDAC1, HAT1, and H3K9Ac in SNUC5 and SNUC5/5-FUR cells determined by Western blot analysis. *Significantly different from SNUC5 cells (p<0.05). **B.** Expression patterns of MLL, H3K4Me3, G9a, and H3K9Me2 in both cells determined by Western blot analysis. TBP antibody was used as loading control for nuclear fractions. *Significantly different from SNUC5 cells (p<0.05). The cells were transfected with non-targeting siRNA (siControl) or siMLL RNA or siHAT1 RNA for 24 h. **C.** Expression patterns of MLL, HAT1, Nrf2, and HO-1 in SNUC5/5-FUR determined by Western blot analysis. *Significantly different from untreated siControl-transfected cells (p<0.05).

As TET-dependent DNA demethylation upregulated Nrf2 expression in SNUC5/5-FUR cells, we investigated the expression levels of histone acetylation- and methylation-related proteins in SNUC5 and SNUC5/5-FUR cells. HDAC1 expression was decreased and HAT1 expression was increased in SNUC5/5-FUR cells compared to SNUC5 cells, resulting in increased H3K9 acetylation (H3K9Ac) (Figure 2A). In addition to histone acetylation, histone methyltransferase MLL and trimethylation of its target protein H3K4 (H3K4Me3) were increased in SNUC5/5-FUR cells compared to SNUC5 cells, while histone methyltransferase G9a and dimethylation of its target protein H3K9 (H3K9Me2) were decreased in SNUC5/5-FUR cells (Figure 2B). Furthermore, siRNA knockdown of MLL in SNUC5/5-FUR cells significantly decreased the expression levels of Nrf2 and its target protein HO-1. Knockdown of HAT1 resulted in a smaller decrease in Nrf2 and HO-1 protein expression than MLL knockdown (Figure 2C). These results led us to focus on MLL to elucidate the relationship between Nrf2 and histone modifications in SNUC5/5-FUR cells.

### MLL and HCF1 increase Nrf2 expression in SNUC5/5-FUR cells

**Figure 3:**
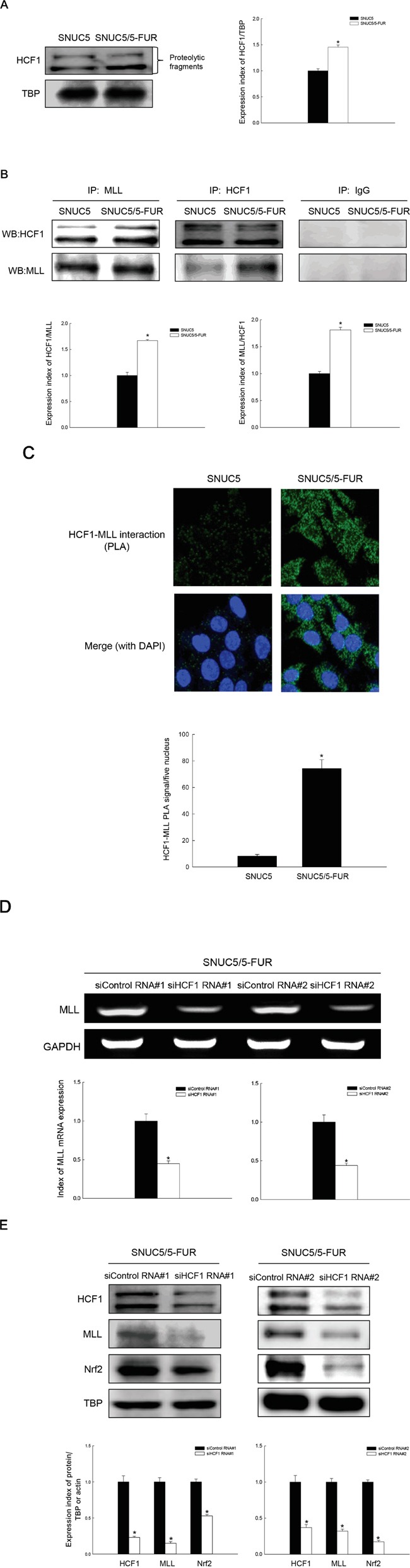
Effect of MLL-HCF1 interaction on histone methylation and Nrf2 expression in SNUC5/5-FUR cells **A.** HCF1 expression was determined by Western blotting. *Significantly different from SNUC5 cells (p<0.05). **B.** Interaction between MLL and HCF1was examined by immunoprecipitation analysis using anti-MLL and anti-HCF1 antibodies followed by Western blotting with anti-HCF1 and anti-MLL antibodies. *Significantly different from SNUC5 cells (p<0.05). **C.** Interaction between MLL and HCF1was assessed by PLA. Each green spot represents for a single interaction (MLL and HCF1) and DNA was stained with DAPI. *Significantly different from SNUC5 cells (p<0.05). The cells were transfected with non-targeting siRNA (siControl) or siHCF1 RNA for 24 h. **D.** Expression pattern of MLL mRNA in SNUC5/5-FUR cells was determined by RT-PCR analysis. *Significantly different from siControl-transfected cells (p<0.05). **E.** Expression pattern of HCF1, MLL, and Nrf2 in SNUC5/5-FUR cells was determined by Western blot analysis. *Significantly different from siControl-transfected cells (p<0.05).

Host cell factor 1 (HCF1), a component of the H3K4 methyltransferase SET1(MLL)/COMPASS complex, is crucial for the integrity of the SET1(MLL)/COMPASS complex [4]. HCF1 undergoes a proteolytic maturation process from HCF1_PRO_ (proform for proteolysis), resulting in stable HCF1_N_ (N-terminal) and HCF1_C_ (C-terminal) subunits [14]. The active form (proteolytic fragments) of HCF1 was increased in SNUC5/5-FUR cells (Figure 3A) and immunoprecipitation (IP) revealed a greater percentage of HCF1 and MLL were found in complex together in SNUC5/5-FUR cells than in SNUC5 cells (Figure 3B). MLL-HCF1 binding measured by proximity ligation assay (PLA) as shown in Figure 3C, was consistent with the IP. A previous study suggested that HCF1 protein functions as a transcriptional switch for the action of MLL [15]. siRNA knockdown of HCF1 led to decreased MLL mRNA and protein in SNUC5/5-FUR cells (Figure 3D and 3E), resulting in decreased Nrf2 protein (Figure 3E). These results suggest that MLL and HCF1 components within the histone methyltransferase, MLL/COMPASS-like complex, increase Nrf2 expression in SNUC5/5-FUR cells.

### OGT increases MLL/COMPASS-like complex-mediated Nrf2 expression

**Figure 4:**
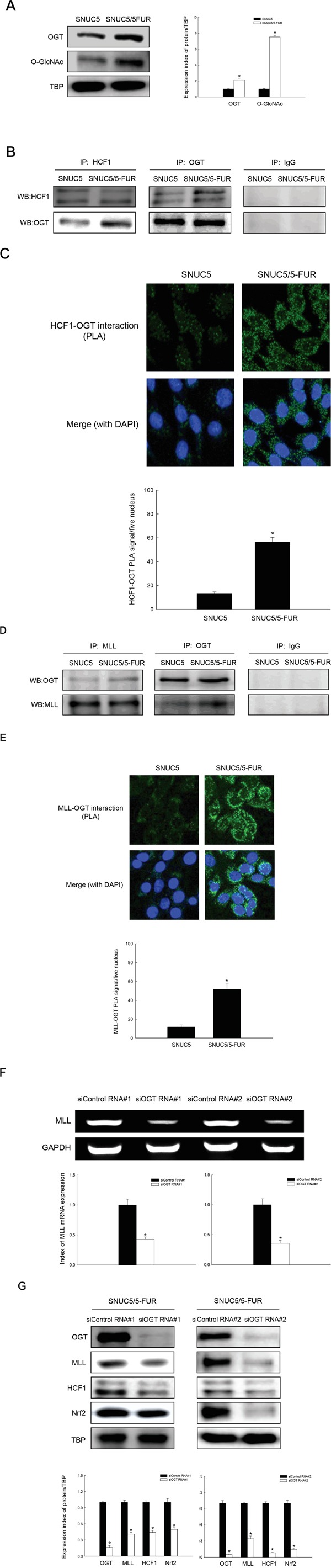
Involvement of OGT in histone methylase MLL/COMPASS-like complex-mediated Nrf2 expression in SNUC5/5-FUR cells **A.** OGT and O-GlcNAc expression was determined by Western blotting. *Significantly different from SNUC5 cells (p<0.05). **B.** Interaction between HCF1 and OGT was examined by immunoprecipitation analysis using anti-HCF1 and anti-OGT antibodies followed by Western blotting with anti-HCF1 and anti-OGT antibodies. **C.** Interaction between HCF1 and OGT was assessed by using PLA. Each green spot represents for a single interaction (HCF1 and OGT) and DNA was stained with DAPI. *Significantly different from SNUC5 cells (p<0.05). **D.** Interaction between MLL and OGT was examined by immunoprecipitation analysis using anti-MLL and anti-OGT antibodies followed by Western blotting with anti-OGT and anti-MLL antibodies. **E.** Interaction between MLL and OGT was assessed by using PLA. Each green spot represents for a single interaction (MLL and OGT) and DNA was stained with DAPI. *Significantly different from SNUC5 cells (p<0.05). The cells were transfected with non-targeting siRNA (siControl) or siOGT RNA for 24 h. **F.** Expression pattern of MLL mRNA in SNUC5/5-FUR cells was determined by RT-PCR analysis. *Significantly different from siControl-transfected cells (p<0.05). **G.** Expression pattern of OGT, MLL, HFC1, and Nrf2 in SNUC5/5-FUR cells was determined by Western blot analysis. *Significantly different from siControl-transfected cells (p<0.05).

Recently, Lazarus et al. suggested that HCF1 is GlcNAcylated and undergoes proteolytic maturation in the active site of O-GlcNAc transferase (OGT) [16]. This OGT link is associated with activation of the regulatory functions of HCF1. SNUC5/5-FUR cells showed high levels of OGT and O-GlcNAcylated proteins, suggesting increased OGT function (Figure 4A). A greater percentage of OGT and HCF1 were found in complex together in SNUC5/5-FUR cells than in SNUC5 cells by IP and PLA (Figure 4B and 4C). Also, more MLL-OGT complex was detected in SNUC5/5-FUR cells than in SNUC5 cells (Figure 4D and 4E), suggesting all three proteins are in a MLL-OGT-HCF1 complex. Recently, it was reported that MLL protein stability is maintained by OGT; depletion of OGT in cells decreased the MLL protein level through ubiquitin/proteasome-dependent proteolytic degradation, whereas ectopic expression of OGT protein suppressed MLL ubiquitylation [17]. siRNA knockdown of OGT led to decreased mRNA and protein levels of MLL in SNUC5/5-FUR cells, resulting in decreased HCF1 and Nrf2 proteins (Figure 4F and 4G).

### TET1-OGT interaction increases Nrf2 expression

**Figure 5:**
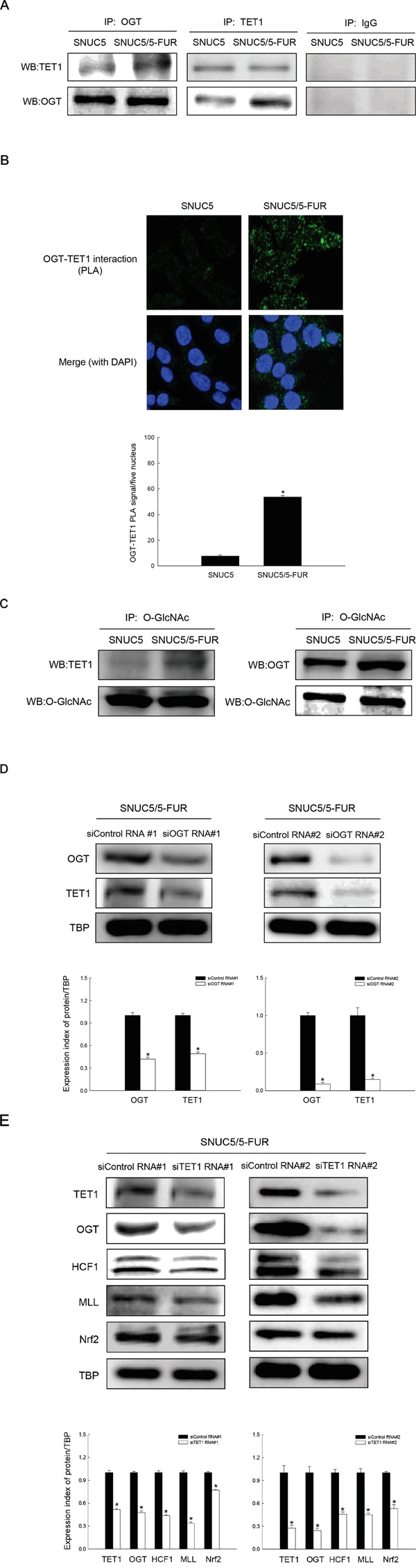
Relation of OGT-TET1 interaction on histone methylation and Nrf2 expression in SNUC5/5-FUR cells **A.** Interaction between OGT and TET1 was examined by immunoprecipitation analyses using anti-OGT and anti-TET1 antibodies followed by Western blotting with anti-TET1 and anti-OGT antibodies. **B.** Interaction between OGT and TET1 was assessed by using PLA. Each green spot represents for a single interaction (OGT and TET1) and DNA was stained with DAPI. *Significantly different from SNUC5 cells (p<0.05). **C.** The O-GlcNAcylated TET1 or O-GlcNAcylated OGT was examined by immunoprecipitation analyses using anti-O-GlcNAc antibody followed by Western blotting with anti-TET1 and anti-OGT antibodies. The cells were transfected with non-targeting siRNA (siControl) or siOGT RNA or siTET1 RNA for 24 h. **D.** Expression pattern of OGT and TET1 in SNUC5/5-FUR cells was determined by Western blot analysis. *Significantly different from siControl-transfected cells (p<0.05). **E.** Expression pattern of TET1, OGT, HCF1, MLL, and Nrf2 in SNUC5/5-FUR cells was determined by Western blot analysis. *Significantly different from siControl-transfected cells (p<0.05).

Recently it was reported that TET-OGT interaction promotes GlcNAcylation and influences H3K4Me3 via histone methyltransferase SET1 (MLL)/COMPASS complex resulting in transcriptional activation [4]. TET1 and OGT form a more stable complex in SNUC5/5-FUR cells than in SNUC5 cells as determined by IP and PLA (Figure 5A and 5B). TET1 GlcNAcylation status was measured using anti-O-GlcNAc antibody. TET1 was detected by Western blot in anti-GlcNAc immunoprecipitates as was OGT because OGT is self GlcNAcylated [6] (Figure 5C). This result suggests that TET1 is associated with OGT in SNUC5/5-FUR cells and is modified by OGT to become GlcNAcylated. To investigate the significance of the TET1-OGT interaction on Nrf2 expression, we knocked down OGT with siRNA oligonucleotides in SNUC5/5-FUR cells. Knockdown of OGT decreased TET1 protein (Figure 5D). Furthermore, knockdown of TET1 led to decreased OGT, HCF1, MLL, and Nrf2 protein in SNUC5/5-FUR cells (Figure 5E). These results suggest that the interaction of TET and OGT is necessary for Nrf2 expression in SNUC5/5-FUR cells.

### TET1-MLL complex increases H3K4Me3 binding to the Nrf2 promoter

**Figure 6:**
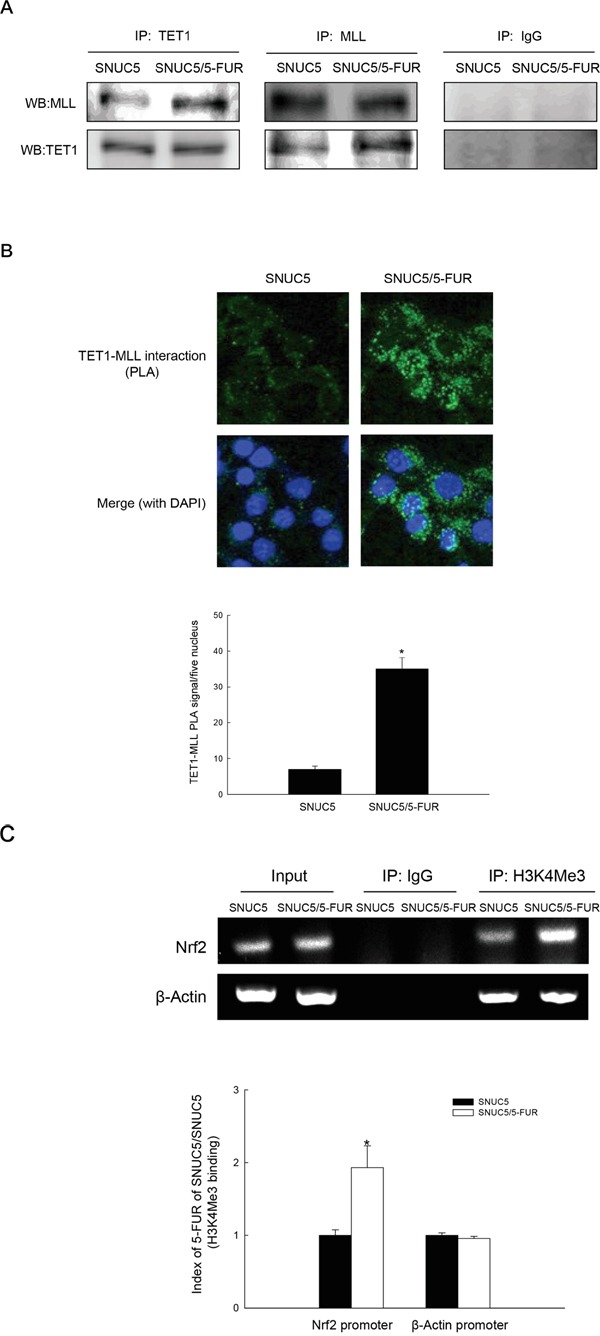
Interaction of TET1 and MLL and binding of H3K4Me3 to Nrf2 promoter in SNUC5/5-FUR cells **A.** Interaction between TET1 and MLL was examined by immunoprecipitation analysis using anti-TET1 and anti-MLL antibodies followed by Western blotting with anti-MLL and anti-TET1 antibodies. **B.** Interaction between TET1 and MLL was assessed by using PLA. Each green spot represents for a single interaction (TET1 and MLL) and DNA was stained with DAPI. *Significantly different from SNUC5 cells (p<0.05). **C.** ChIP analysis was performed using anti-H3K4Me3 antibody and primers to amplify the Nrf2 or β-actin promoter region. Bands indicate level of H3K4Me3 in the Nrf2 or β-actin promoter region. Input represents amplification of total DNA from whole cell lysates. *Significantly different from SNUC5 cells (p<0.05).

Because TET1 knockdown decreased MLL in SNUC5/5-FUR cells (Figure 5E), we assessed whether TET1 interacts with MLL. Immunoprecipitation and PLA revealed that the binding between TET1 and MLL was greater in SNUC5/5-FUR cells than in SNUC5 cells (Figure 6A and 6B). To elucidate whether Nrf2 upregulation is directly induced by the epigenetic modifications, chromatin immunoprecipitation (ChIP) assay was used to quantify the H3K4Me3 binding to the Nrf2 promoter. As shown in Figure 6C, H3K4Me3 binding to the Nrf2 promoter was significantly increased in SNUC5/5-FUR cells, while H3K4Me3 binding to the β-actin promoter, unrelated to 5-FU resistance, was unchanged.

### 5-FU affects histone methyltransferase through reactive oxygen species (ROS) production

**Figure 7:**
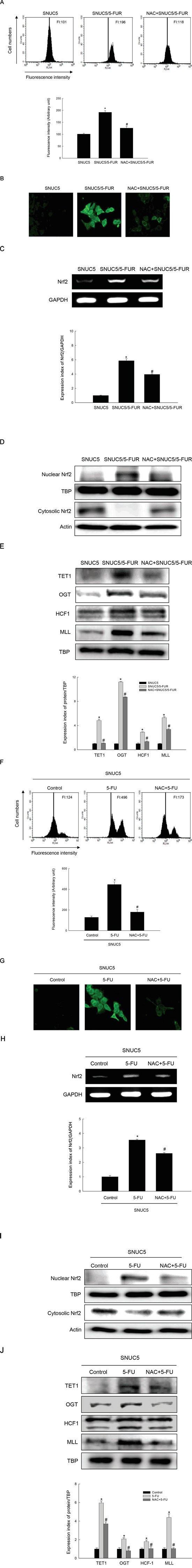
5-FU-produced ROS involved in histone methyltransferase-mediated Nrf2 expression SNUC5/5-FUR cells were pre-treated with 500 μM NAC, plated, and incubated for 48 h. Then, cells were treated with 10 μM DHR123 and incubated for 30 min at 37°C. The intracellular ROS were detected by **A.** flow cytometry and **B.** confocal imaging. FI indicates the fluorescence intensity of DHR-123. *Significantly different from SNUC5 cells (p<0.05), and ^#^significantly different from SNUC5/5-FUR cells (p<0.05). **C.** Expression pattern of Nrf2 mRNA was determined by RT-PCR analysis. *Significantly different from SNUC5 cells (p<0.05), and ^#^significantly different from SNUC5/5-FUR cells (p<0.05). **D.** Expression pattern of nuclear and cytosolic Nrf2 protein was determined by Western analysis. **E.** Expression patterns of TET1, OGT, HCF1, and MLL in NAC pre-treated SNUC5/5-FUR cells determined by Western blot analysis. *Significantly different from SNUC5 cells (p<0.05), and ^#^significantly different from SNUC5/5-FUR cells (p<0.05). Also, SNUC5 cells were pre-treated with 500 μM of NAC and incubated for an additional 1 h at 37°. Cells were then treated with 140 μM 5-FU for 48 h. 10 μM of DHR123 was added to plate and incubated for an additional 30 min at 37°C. **F.** The intracellular ROS level was detected by flow cytometry and **G.** confocal imaging. FI indicates the fluorescence intensity of DHR123. *Significantly different from SNUC5 cells (p<0.05), and ^#^significantly different from 5-FU-treated SNUC5 cells (p<0.05). **H.** Expression pattern of Nrf2 mRNA was determined by RT-PCR analysis. *Significantly different from SNUC5 cells (p<0.05), and ^#^significantly different from 5-FU-treated SNUC5 cells (p<0.05). **I.** Expression pattern of nuclear and cytosolic Nrf2 protein was determined by Western analysis. **J.** The expression of TET1 and interaction proteins detected by Western blot analysis. *Significantly different from SNUC5 cells (p<0.05), and ^#^significantly different from 5-FU-treated SNUC5 cells (p<0.05).

Persistent oxidative stress induces genomic instability, carcinogenesis, and anticancer drug resistance [18]. Recently, we showed that SNUC5/5-FUR cells undergo oxidative stress due to 5-FU-induced intracellular ROS, resulting in activation of TET-dependent DNA demethylation and induction of Nrf2 expression [13]. Therefore, we investigated the effect of 5-FU-induced ROS on H3K4Me3-related proteins. SNUC5/5-FUR cells showed higher intracellular ROS levels than SNUC5 cells detected by using flow cytometry and confocal microscopy after staining of DHR123 (Figure 7A and 7B), and increased Nrf2 mRNA (Figure 7C) and nuclear Nrf2 protein (Figure 7D). SNUC5/5-FUR cells exhibited higher TET1, OGT, HCF1, and MLL than SNUC5 cells (Figure 7E). Treatment with the antioxidant N-acetylcystein (NAC) reduced ROS and expression of Nfr2, TET1, OGT, HCF1, and MLL protein (Figures 7A–7E). These results suggest that SNUC5/5-FUR cells are exposed to oxidative stress condition.

**Figure 8:**
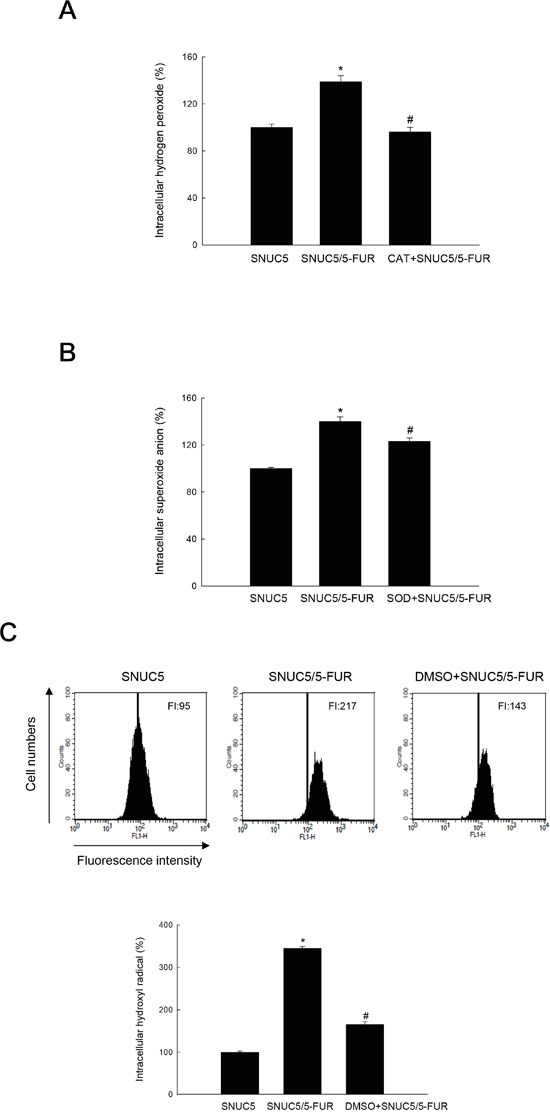
Detection of H_2_O_2_, O_2_^−^, and HO· in SNUC5 and SNUC5/5-FUR cells SNUC5/5-FUR cells were pre-treated with specific ROS scavengers, CAT for H_2_O_2_ at 0.5 U/ml, SOD for O_2_^−^ at 30 U/ml, and 2% DMSO for HO· radical, and incubated for 48 h. Then cells were treated with ROS probes (25 μM of DCF-DA for H_2_O_2_, 10 μM of DHE for O_2_^−^ and 10 μM of HPF for HO·) and incubated for 30 min at 37°C. **A.** The H_2_O_2_ level and **B.** O_2_^−^ level were detected by fluorescence spectroscopy. *Significantly different from SNUC5 cells (p<0.05), and ^#^significantly different from SNUC5/5-FUR cells (p<0.05). **C.** HO· radical was detected by flow cytometry. FI indicates the fluorescence intensity. *Significantly different from SNUC5 cells (p<0.05), and ^#^significantly different from SNUC5/5-FUR cells (p<0.05).

We investigated whether 5-FU induces specific ROS by detecting their intracellular levels. 5-FU treatment of SNUC5 cells increased ROS levels (Figure 7F and 7G), Nrf2 mRNA (Figure 7H), and nuclear Nrf2 protein level (Figure 7I). 5-FU treatment also increased TET1, OGT, HCF1, and MLL protein (Figure 7J). NAC treatment reduced all of these effects (Figures 7F–7J). To identify the nature of 5-FU-mediated ROS, intracellular H_2_O_2_, O_2_^−^, and HO· levels were measured, using DCF-DA, DHE and HPF assays, respectively. The H_2_O_2_ level by DCF-DA assay was significantly higher in SNUC5/5-FUR cells than in SNUC5 cells; however, catalase (CAT, a specific scavenger of H_2_O_2_) treatment decreased the H_2_O_2_ level in SNUC5/5-FUR cells (Figure 8A). Additionally, SNUC5/5-FUR cells exhibited increased O_2_^−^ level, detected by a DHE fluorescence dye, compared to SNUC5 cells; the presence of superoxide dismutase (SOD, a specific scavenger of O_2_^−^) significantly reduced the O_2_^−^ level in SNUC5/5-FUR cells (Figure 8B). Next, we assessed intracellular HO· radical generation in SNUC5 and SNUC5/5-FUR cells. HPF fluorescence intensity (FI) was significantly higher in SNUC5/5-FUR cells compared to SNUC5 cells. Addition of dimethyl sulfoxide (DMSO), an HO· radical scavenger, significantly decreased HO· (Figure 8C). These results suggest that 5-FU induces various ROS radicals including H_2_O_2_, O_2_^−^, and HO· and the effect of 5-FU on histone methylation is mediated by its induction of these ROS.

## DISCUSSION

Recently, we demonstrated that TET1-mediated Nrf2 expression mediates 5-FU resistance in Colon cancer cells [13]. In the present study, we showed that expression of Nrf2 is upregulated via MLL-mediated histone modification and DNA demethylation.

Di- or trimethylation of histone H3 at lysine 4 (H3K4) is found at actively transcribed genes [19] and is mediated by the MLL/COMPASS-like complexes [20, 21]. MLL/COMPASS-like complexes contain several well-characterized, common subunits; HCF1, ASH2, WDR5 and RbBP5, which are conserved between yeast and humans [22–24]. These subunits are thought to constitute a common MLL/COMPASS-like complex that forms a platform that mediates the MLL enzyme interaction with H3K4 substrate [22, 24–26]. H3K4 methylation has been associated with a removal of repressive H3K9 methylation or H3K27 methylation by MLL/COMPASS-associated histone demethylases [27–29]. Observations that the deposition of activating methylation events on H3K4 correlates with the removal of silencing methylation events on H3K27 have important implications for the regulation of bivalent genes, including cytokine-inducible genes of the immune system.

Our data show that HCF1, an integral component of the MLL/COMPASS-like complex, is highly expressed in SNUC5/5-FUR cells compared to SNUC5 cells and exists in stable complex with MLL. In addition, HCF1 interacts with and is modified by OGT [30], which adds O-GlcNAc to serine and threonine residues [31]. HCF1 undergoes proteolytic maturation process at six centrally located HCF-1_PRO_-repeat sequences resulting in stably associated HCF1_N_ and HCF1_C_ subunits. Deplus et al. reported that this GlcNAcylated, stable HCF1 is necessary for the integrity of the SET1/COMPASS complex [4]. Also, our data show that OGT is highly expressed in SNUC5/5-FUR cells compared to SNUC5 cells and exists in complex with HCF1 and MLL. The SET1/COMPASS complex trimethylates lysine 4 of histone H3 (H3K4Me3). H3K4Me3 in the nucleosome promotes transcription of nearby DNA. OGT and TET2/3 co-localize with CpG islands and transcription start sequences and TET2/3-OGT interacts with all components of the SET1/COMPASS complex [4]. By reducing TET2 and TET3 expression levels through RNA interference, reduced TET expression leads to decreased OGT activity, reduced HCF1 GlcNAcylation, and, lower levels of H3K4Me3. In other words, less TET2/3 protein resulted in less HCF1 GlcNAcylation, fewer SET1/COMPASS complexes, less H3K4Me3, and lower transcriptional activity [4]. We show that TET1 interacts with OGT and undergoes O-GlcNAcylation, and TET1-mediated Nrf2 expression is dependent on OGT. OGT posttranslationally modifies protein substrates by adding GlcNAcyl moieties to serine/threonine residues [32]. O-GlcNAcylation is crucial to diverse biological processes including nutrient and growth factor sensing, cell cycle progression, and stress response [6, 33]. Vella et al. demonstrated that OGT preferentially associates with TET1 at gene promoters in close proximity to CpG-rich transcription start sites and OGT-mediated O-GlcNAcylation stabilizes TET1 activity at promoters [9].

**Figure 9:**
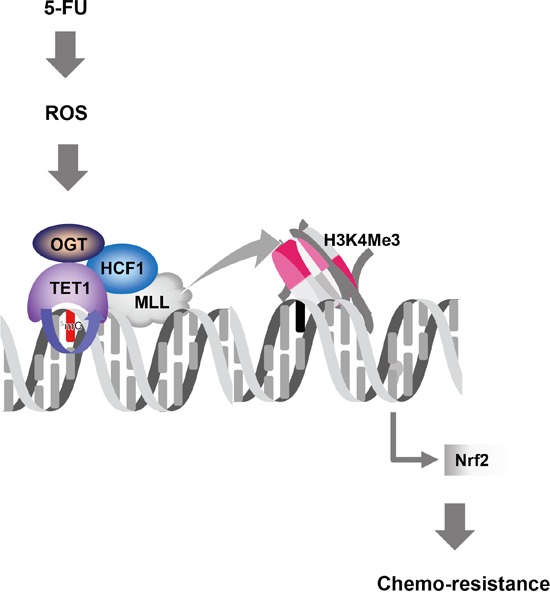
The proposed diagram for DNA and histone modification mechanism of resistance to 5-FU in colon cancers 5-FU induces oxidative stress via generation of ROS. Then, oxidative stress-activated TET1 protein recruits OGT to the Nrf2 promoter, and OGT GlcNAcylates HCF1, a component of MLL/COMPASS-like complex. This H3K4Me3 methyltransferase complex methylates H3K4, resulting in transcriptional activation of Nrf2, and leads to chemo-resistance.

SNUC5/5-FUR cells with higher TET1 and OGT complex showed increased O-GlcNAcylated protein level compared to SNUC5 cells. Deplus et al. demonstrated that TET2 and TET3 regulate GlcNAcylation and H3K4 methylation through OGT and SET1/COMPASS, and TET1 localizes primarily at regions high in H3K4Me3 [4, 10, 11]. This suggests that in addition to reducing DNA methylation, the TET-OGT interaction might recruit proteins required to establish a high H3K4Me3 chromatin environment. Depletion of OGT reduces TET1, whereas ectopic expression of wild-type OGT increases TET1 levels. Mutation of the putative O-GlcNAcylation site on TET1 decreases O-GlcNAcylation and the level of the TET1 protein. These results suggest that O-GlcNAcylation increases TET1 protein expression and that TET1-mediated 5-hydroxylmethylcytosine modification is controlled by OGT. Our data confirm that OGT expression is increased in SNUC5/5-FUR cells and that TET1 interacts with OGT. Recent studies also found that OGT interacts with TET family proteins; interestingly, TET proteins appear to recruit OGT to CpG rich promoters in embryonic stem cells [4, 9, 34]. Knockdown of TET1 or TET2 led to loss of OGT from chromatin [9, 34]; knockdown of TET2 also reduced O-GlcNAc modification of histones [34]. Moreover, interaction with TET2/3 stimulates OGT activity toward HCF1 [4]. Given this connection between OGT, TET proteins, and HCF1, it is likely that TET proteins are key factors in recruiting SET or MLL complexes to CpG islands. In line with this notion, OGT- and TET-binding sites overlap to a large extent with H3K4Me3 sites at promoters [4, 9]. Both OGT activity and its interaction with TET2/3 promoted chromatin association of SET complexes and H3K4 methylation in HEK293 cells [4]. Moreover, previously, we demonstrated that 5-FU treatment stimulates ROS production and is associated with TET1 activation [13]. Our data show that 5-FU treatment generated various ROS such as H_2_O_2_, O_2_^−^and HO·. In summary, Nrf2 transcription factor in 5-fluorouracil-resistant colon cancer cells is upregulated via interaction of DNA demethylase and histone methyltransferase through oxidative stress (Figure 9).

## MATERIALS AND METHODS

### Cell culture

SNUC5 colon cancer cells (KCLB no. 0000C5) were obtained from the Korean Cell Line Bank (Seoul, Republic of Korea) and cultured at 37°C in a 5% CO_2_ atmosphere using RPMI 1640 medium with 10% heat-inactivated FBS. The chemo-resistant SNUC5 (SNUC5/5-FUR, SNUC5/OXTR) cells were selected from the parental wild type SNUC5 by chronic exposure to 5-FU and OXT (Sigma-Aldrich Co., St. Louis, MO, USA), respectively, in intermittent dosage schedules at sufficient intervals to permit the expression of the resistance phenotypes. The cells were exposed to the drugs starting from 1 × IC_50_ (5-FU 17.5 μM and OXT 7.14 μM), and the concentrations were escalated at an increasing rate of 50%, and then finally cultured at fixed concentrations over a level of 10 times the IC_50_ of 5-FU and OXT, respectively. Cells were cultured in a fixed 5-FU (140 μM) and OXT (7.14 μM) concentration for all subsequent experiments [35]. Human ovarian cancer cells A2780 and its cisplatin resistant derivative A2780/CR were obtained from Sigma-Aldrich Co and were cultured in RPMI-1640 supplemented with 10% FBS and 1% penicillin/streptomycin. To maintain resistance, A2780/CR cells were treated with 1 μM cisplatin after every third passage.

### Western blot analysis

Cells were harvested and lysed on ice in 1 ml of lysis buffer (10 mM Tris-HCl, pH 7.9, 10 mM NaCl, 3 mM MgCl_2_, and 1 % NP-40) for 4 min. After centrifugation for 10 min at 3,000 × g, the pellets were re-suspended in 50 μl of extraction buffer (20 mM HEPES, pH 7.9, 20% glycerol, 1.5 mM MgCl_2_, 0.2 mM EDTA, 1 mM DTT, and 1 mM PMSF), incubated on ice for 30 min, and centrifuged at 13,000 × g for 5 min. Following measurement of the protein concentration, supernatants were stored at −70°C. Aliquots of the lysates (40 μg of protein) were boiled for 5 min and electrophoresed on a 10% SDS-polyacrylamide gel. Proteins were transferred onto nitrocellulose membranes, which were subsequently incubated with primary antibodies, followed by secondary immunoglobulin-G-horseradish peroxidase conjugates (Pierce, Rockford, IL, USA). The primary antibodies were as follows: TET1 (Abcam, Cambridge, MA, USA; ab191698), HDAC1 (Santa Cruz Biotechnology, Santa Cruz, CA, USA; sc-7872), HAT1 (Santa Cruz Biotechnology; sc-366092), H3K9Ac (Cell Signaling Technology, Danvers, MA, USA; #9671), MLL (Active Motif, Carlsbad, CA, USA; #61295), H3K4Me3 (Abcam; ab8580), G9a (Santa Cruz Biotechnology; sc-22877), H3K9Me2 (Abcam; ab1220), HO-1 (Cell Signaling Technology; #5853), HCF1 (Novus Biologicals, Littleton, CO, USA; NB-68209), Nrf2 (Santa Cruz Biotechnology; sc-722); OGT (Santa Cruz Biotechnology; sc-32921), O-GlcNAc (Santa Cruz Biotechnology; sc-59623); TBP (Abcam; ab818) and actin (Santa Cruz Biotechnology;sc-1616). Protein bands weredetected using an enhanced chemiluminescence Western blotting detection kit (Amersham, Little Chalfont, UK). The protein bands were visualized using a luminescent image analyzer and quantified.

### Transient transfection of small interfering RNAs (siRNAs)

Cells were seeded at a density of 1.5 × 10^5^ cells/ml in 24 well plates and allowed to reach approximately 50% confluence on the day of transfection. Cells were transfected with 10–50 nM of a mismatched siRNA control (siControl) or siRNAs against MLL, HAT1, TET1, HCF1, or OGT (Table 1) using Lipofectamine RNAiMax (Invitrogen, Carlsbad, CA, USA) according to the manufacturer's instructions. At 24 h after transfection, the cells were examined by Western blotting.

**Table 1:** Information on siRNAs (5′→3′) used

siMLL RNA#1	sc-38039
siMLL RNA #2	Sense : GUCACAGUAGGUGAUCCUU Antisense : AAGGAUCACCUACUGUGAC
siHAT1 RNA#1	sc-37948
siHAT1 RNA #2	Sense : GAAAGAUGGCACUACUUUC Antisense : GAAAGUAGUGCCAUCUUUC
siHCF1 RNA#1	sc-37996
siHCF1 RNA #2	Sense : CCGUCCCUGACUAUAACCA Antisense : UGGUUAUAGUCAGGGACGG
siOGT RNA#1	sc-40780
siOGT RNA #2	Sense : GAUUAACCGAGGACAGAUU Antisense : AAUCUGUCCUCGGUUAAUC
siTET1 RNA#1	sc-90457
siTET1 RNA #2	Sense : CAGUGUAACCAGCACAGUU Antisense : AACUGUGCUGGUUACACUG
siControl RNA #1	sc-37007
siControl RNA #2	Sense : UUCUCCGAACGUGUCACGUTT Antisense : ACGUGACACGUUCGGAGAATT

### Immunoprecipitation method

Cells were lysed in 0.5% NP40, 10 mM, Tris-HCl (pH 8.0), 150 mM NaCl, and 5 mM MgCl_2_. Lysates were incubated overnight at 4°C with HCF1, MLL, TET1, OGT, or IgG antibody. Immune complexes were collected with protein A/G PLUS-beads (Santa Cruz Biotechnology) overnight at 4°C, and washed with immunoprecipitation buffer. Equal amounts of the precipitates were separated by SDS-polyacrylamide gel electrophoresis, followed by Western blot analysis with antibodies specific for HCF1, MLL, TET1, and OGT.

### Proximity ligation assay (PLA)

The mouse/rabbit red starter Duolink kit (Sigma-Aldrich Co.) was used for this experiment. The cells were seeded at 1.5 × 10^3^ cells/ml in a 4 well chamber slide (Thermo Fisher, Scoresby, Victoria, Australia). After washing with PBSCM (PBS, 1 mM CaCl_2_, 1 mM MgCl_2_) three times, the cells were fixed with cold 3% paraformaldehyde (PFA) for 15 min at room temperature (RT). The fixed cells were then washed with 50 mM NH_4_Cl to quench the PFA followed by a PBSCM wash and permeabilized with 0.1% saponin in PBSCM for 15 min at RT. After permeabilization the cells were incubated in the blocking buffer (provided with the kit) overnight at 37°C in a humidified chamber and then incubated with primary antibodies: rabbit anti-TET1 (1:500), goat anti-TET1 (1:100), mouse anti-OGT (1:500), rabbit anti-MLL (1:1000) or goat anti-HCF-1 (1:100) diluted in fluorescence dilution buffer (FDB) (5% fetal calf serum, 5% normal donkey serum, 2% bovine serum albumin in PBSCM, pH7.6) for 2 h at RT. For the rest of the protocol the manufacturer's instructions were followed. Briefly, the cells were washed in buffer A (supplied with the kit) 3 times for 15 min and incubated with the PLA probes for 1 h at 37°C in a humid chamber. This was followed by a 10 min wash and a 5 min wash in buffer A. The ligation reaction was carried out at 37°C for 1 h in a humid chamber followed by a 10 min wash and 5 min wash in buffer A. The cells were then incubated with the amplification mix for 2 h at 37°C in a darkened humidified chamber. After washing with 1 × buffer B (supplied with the kit) for 10 min followed by 1 min wash with 0.01 × buffer B the cells were mounted using the mounting media supplied with the kit.

### Reverse transcription-polymerase chain reaction (RT-PCR)

Total RNA was isolated using Trizol (GibcoBRL, Grand Island, NY, USA). PCR conditions for Nrf2, MLL, and housekeeping gene, GAPDH, were: 35 cycles of 94°C for 45 sec; 53°C for 45 sec; and 72°C for 60 sec. The primer pairs (Bioneer, Daejeon, Republic of Korea) were as follows (forward and reverse, respectively): Nrf2, 5′-GGCCCGGACTCTTGC-3′ and 5′-GGCGGCCCTGTTCC-3′; MLL, 5′-GACAAAGGG AATGGCAAGAA-3′ and 5′-TATCCTGCTGCTCAGC CTCT-3′ and GAPDH, 5′-AAGGTCGGAGTCAAC GGATTT-3′, and 5′-GCAGTGAGGGTCTCTCTCCT-3′. Amplified products were resolved by 1% agarose gel electrophoresis, stained with ethidium bromide, and photographed under ultraviolet light.

### Chromatin immune-precipitation (ChIP) assay

The ChIP assay was performed using the Simple ChIP™ enzymatic chromatin IP kit (Cell Signaling Technology) according to the manufacturer's protocol with slight modifications. Briefly, cells were cross-linked by addition of 1% formaldehyde. Chromatin was prepared and digested with nuclease for 12 min at 37°C. ChIP was performed with an antibody against tri-methylated histone H3 lysine 4 (H3K4Me3) and mouse IgG. Antibodies were added to the chromatin digests and incubated with constant rotation overnight at 4°C. ChIP-grade protein G magnetic beads were then added to capture the immune complexes. The beads were washed and the immune-precipitates were eluted with ChIP elution buffer. The cross-links were reversed by incubation at 65°C for 30 min. Proteinase K was added and samples were incubated at 65°C for 2 h. The immunoprecipitated DNA fragments were purified using spin columns. DNA that was recovered from the immunoprecipitated complex was subjected to 35 cycles of PCR. PCR of the Nrf2 promoter region across the H3K4Me3 binding sites within −505 to −254 of the Nrf2 promoter was performed: sense, 5′-AGAGAAAGTAAGCTCTGCAGC-3′; antisense, 5′-CTGGCAGTGGTTTTGCTATTT-3′ for the Nrf2 (H3K4Me3-binding site) gene. PCR of the β-actin promoter region across the H3K4Me3 binding sites within −1541 to −1392 of the β-actin promoter was assessed: sense, 5′-GCCCTTAAGGCTGAGAAGGT-3′ and antisense, 5′- GTAGAGACGGGGTTTCACCA-3′ for the β-actin (H3K4Me3-binding site) gene. The PCR products were separated on 2% agarose gels, and DNA bands were visualized using the ImageJ program(NIH, Bethesda, MD, USA).

### Reactive oxygen species (ROS) assay

Cells were seeded in 6 well plates at a density of 3 × 10^5^ cells/ml. After 24 h of incubation at 37°C, the cells were treated for 24 h with 140 μM 5-FU. Cells were treated with 20 μM dihydrorhodamine (DHR) 123, and the DHR123 fluorescence was detected using a flow cytometer (Becton Dickinson, Mountain View, CA, USA) and analyzed using Cell Quest software. For image analysis of the generation of intracellular ROS, cells were seeded on a coverslip-loaded 6-well plate at a density of 2 × 10^5^ cells/ml. After addition of 20 μM DHR123 to each well and incubation for an additional 30 min at 37°C, plates were washed with PBS, and stained cells were mounted onto a microscope slide in mounting medium (DAKO, Carpinteria, CA, USA). Microscopic images were analyzed using the Laser Scanning Microscope 5 PASCAL program (Carl Zeiss, Jena, Germany) on a confocal microscope.

### Intracellular hydrogen peroxide (H_2_O_2_) assay

The DCF-DA method was used to detect the levels of intracellular H_2_O_2_. Cells were seeded in a 96 well plate at 2 × 10^4^ cells/ml. Sixteen hours after plating, cells were treated with catalase (CAT, a specific scavenger of H_2_O_2_) at 0.5 U/ml. After 48 h, 25 μM of DCF-DA solution was added. After the addition of 25 μM of DCF-DA solution for 10 min, 2′,7′-dichlorofluorescein fluorescence was detected using a Perkin Elmer LS-5B spectrofluorometer. Fluorescence readings were detected using a plate reader with exit/emission at 485 nm/535 nm.

### Intracellular superoxide anion (O_2_^−^) assay

To investigate the superoxide anion inside the cells, the fluorescent probe dihydroethidium (DHE) was used. DHE reacts primarily with O_2_^−^ to form an oxidized fluorescent product. After entering the cell, this probe is oxidized to ethidium [36]. The cells were seeded in a 96 well plate at 2 × 10^4^ cells/ml. Sixteen hours after plating, cells were treated with superoxide anion dismutase (SOD, a specific scavenger of O_2_^−^) at 30 U/ml. After 48 h, 10 μM DHE was added. The plate was incubated for 30 min in a dark condition. Fluorescence readings were detected using a plate reader with exit/emission at 510 nm/595 nm. The fluorescence was detected using a Perkin Elmer LS-5B spectrofluorometer.

### Intracellular hydroxyl radical (HO·) assay

The fluorescent dye 3-(p-hydroxyl-phenyl) fluorescein (HPF) was used at a concentration of 5 μM to assess the hydroxyl radical level inside the cells. Cells were seeded in 6 well plates at the density of 3 × 10^5^ cells/ml. Sixteen hours after plating, cells were treated with dimethyl sulfoxide (DMSO), a hydroxyl radical scavenger, at a final concentration of 2%. After 48 h, cells were treated with 5 μM of HPF in a dark condition, and the fluorescence was detected using a flow cytometer (Becton Dickinson, Mountain View, CA, USA) and analyzed by the Cell Quest software. Fluorescence readings were detected using a plate reader with exit/emission at 485 nm/528 nm.

### Statistical analysis

All measurements were performed in triplicate, and values are expressed as the mean ± the standard error of the mean (SEM). The results were examined using analysis of variance (ANOVA) and Tukey's test to determine pairwise differences. A p<0.05 was considered significant.
